# Glycolytic Metabolites Are Critical Modulators of Oocyte Maturation and Viability

**DOI:** 10.1371/journal.pone.0077612

**Published:** 2013-10-22

**Authors:** Lloyd Berger, Andrew Wilde

**Affiliations:** The Department of Molecular Genetics and the Department of Biochemistry, University of Toronto, Toronto, Ontario, Canada; UC Irvine, United States of America

## Abstract

The maturation of an oocyte into an egg is a key step in preparation for fertilization. In *Xenopus*, oocyte maturation is independent of transcription, being regulated at the level of translation and post-translational modifications of proteins. To identify factors involved in the maturation process we used two-dimensional differential gel electrophoresis to compare the proteome of oocytes and eggs. Protein abundance changes were observed in multiple cellular pathways during oocyte maturation. Most prominent was a general reduction in abundance of enzymes in the glycolytic pathway. Injection into oocytes of the glycolytic intermediates glyceraldehyde-3-phosphate, phosphoenolpyruvate and glucose-6-phosphate prevented oocyte maturation. Instead, these metabolites stimulated ROS production and subsequent apoptosis of the oocyte. In contrast, all other metabolites tested had no effect on oocyte maturation and did not induce apoptosis. These data suggest that a subset of glycolytic metabolites have the capacity to regulate oocyte viability.

## Introduction

 The maturation of an oocyte into a fertilizable egg is the final stage of oogenesis that must be carefully controlled to coincide with the reproductive cycle of the animal. During maturation the *Xenopus* oocyte goes through many changes [1,2] including changes in metabolism [3]. Disruption of metabolic pathways can have significant consequences on the viability of matured oocytes, eggs and early development [4–6]. Consequently, the viability and survival of oocytes and eggs may be dependent, in part, on the metabolites derived from their nutrient stockpiles or neighboring cells [7]. 

*Xenopus* oocytes grow and mature through a series of defined stages until they become arrested at stage VI in prophase I of meiosis [8]. The VI oocyte can be stored within the female for months. The final stage of oocyte maturation is stimulated by the hormone progesterone, or a closely related metabolite [9,10]. The oocyte then progresses through the final stages of meiosis, becoming arrested at metaphase II of meiosis. Subsequently, it is laid as an egg to be externally fertilized. *Xenopus* oocyte maturation is not regulated by transcription. Instead, oocyte maturation is regulated at the level of translation and through the post-translational modification of proteins [1,2]. The resulting changes to the proteome, both abundance and protein modifications are responsible for the signaling pathways that mature the oocyte.

 The energy required for oocyte maturation comes mainly from oxidative phosphorylation fed by amino acids rather than glucose from glycogen breakdown [11–14]. However, a small proportion of glucose (approximately 5% of glucose) is metabolized through the pentose phosphate pathway (PPP) [15]. One role of the PPP is the production of NADPH. NADPH regulates the redox equilibrium of a cell to maintain enzyme activity and prevent cellular damage [16]. Cellular redox status is determined by the interplay between reactive oxygen species (ROS) production and ROS sequestration. ROS is produced through the electron transport chain and the NADPH oxidase system, whereas ROS is sequestered via the glutathione and thioredoxin systems, which utilize NADPH as the source of reducing power [17]. As the elevation of ROS levels in *Xenopus laevis* oocytes correlates with increased apoptosis [5,6], it is critical to tightly regulate metabolism in order to maintain oocyte and egg integrity.

To further understand the molecular events of *Xenopus* oocyte maturation we compared the proteome of stage VI oocytes with *in situ* progesterone-matured oocytes using two-dimensional differential gel electrophoresis (2D-DIGE). We identified changes to several pathways, including the glycolytic pathway. Further analysis found that altered glycolytic metabolite levels could influence oocyte viability, suggesting that there maybe be more exquisite regulation of oocyte viability through metabolite levels than was previously proposed.

## Methods and Materials

### Reagents

 All reagents used for treatment of, or injection into oocytes, were obtained from Sigma-Aldrich. Antibodies were obtained from the following companies; anti-cytochrome C (Stressgen), anti-p44/42 extracellular regulated kinase and anti-phospho p44/42 extracellular regulated kinase (Cell Signaling Technology), α-tubulin (Sigma). The reagent for detection of reactive oxygen species was 2’,7’-dichlorodihydrofluorescein diacetate (H_2_DCFDA) obtained from Invitrogen, Molecular Probes.

### Animals and oocytes

 Sexually mature *Xenopus laevis* females were obtained from Nasco (Fort Atkinson, WI). Frogs were housed at 18°C on a 12-hour light: 12-hour night cycle. Animal care protocols were carried out in strict accordance with the recommendations of The Canadian Council on Animal Care, the requirements under the Animals for Research Act and the University of Toronto Animal Care policies and guidelines. The protocol was approved by the University of Toronto animal care committee on the Ethics of Animal Experiments (protocol number: 20006884). All surgery was performed under 3-aminobenzoic ethyl ester (tricaine) anesthesia, and all efforts were made to minimize suffering. To obtain oocytes the frogs were anesthetized in a bath containing 2% ethyl 3-aminobenzoate methanesulfonate and a portion of ovary was removed from an abdominal incision. Mature stage VI oocytes were obtained by treatment of ovary with collagenase (Worthington, type 2; 1 mg/ml) dissolved in OR2 buffer (5 mM Hepes pH 7.6, 82.5 mM NaCl, 2.5mM KCl, 1 mM MgCl_2_, 1 mM CaCl_2_) for 2-3 hours. After collagenase treatment oocytes were allowed to recovery for at least 16 hours. Stage VI oocytes were selected and stored in OCM media (60% Leibovitz media (Gibco), 0.04% bovine serum albumin (BioShop), 50 µg/ml gentamycin (Sigma-Aldrich), penicillin (100 U/ml) and streptomycin (10 U/ml)). Oocytes were allowed to recover for at least 16 hours after collagenase treatment prior to being used in experiments. For experimental procedures oocytes were incubated in either OCM or OR2 media as indicated. For extended storage (up to 4 weeks) oocytes were kept at 4°C. Maturation and apoptosis were scored based on the characteristic white spot formation (WSF) at the animal pole of the oocyte. Maturation was confirmed by dissection of oocytes fixed in 10% trichloroacetic acid to confirm germinal vesicle breakdown.

### 2D: DIGE

 After isolation, stage VI oocytes from a single frog were split into two groups. One group remained in OCM media, the other OCM media plus 10µg/ml progesterone. Both groups were incubated at 22°C for 6 hours, a time when virtually all oocytes incubated with progesterone had developed a white spot. Oocytes were then placed in a homogenizer, allowed to settle and excess media removed by aspiration. An equal volume of lysis buffer (7M Urea, 2M Thiourea, 30mM Tris pH to 8.5 and 4% CHAPS) was added and the oocytes homogenized. The lysates were incubated on ice for 1 hour, then centrifuge at 10000g for 20 mins to remove insoluble material. 50µg of each lysate was then labeled with Cy3 or Cy5 CyDye DIGE fluors dyes according to the manufacturers instructions (GE Healthcare). After labeling the samples were separated by 2D electrophoresis in the presence of a third sample, the internal standard that comprised of an equal concentration of oocyte and progesterone matured oocyte lysates labeled with Cy2 CyDye DIGE fluor. The final SDS-PAGE gel was then imaged on a Typhoon scanner and the fluorescence intensity of the protein spots was measured. This was repeated using oocytes from 3 different frogs in total. As the frogs are not from inbred isogenic lines, this reflects an analysis of genetically distinct individuals. Although this approach may have led to fewer differences being discovered than if isogenic lines could have been used, those differences that are discovered are likely to be the best conserved changes to the proteome.

 Using the DeCyder software (GE Health Sciences) the scanned images from the gels were first aligned using the differential in-gel analysis module. From the aligned gel images 1096 protein spots could be matched across the gels. Next a quantitative comparison of spots in the different gels was made using the biological variance analysis module (BVA) of DeCyder. Spots (approx. 1 mm diameter) were removed from Cy-dye labeled samples using an Ettan robotic spot picker (GE Health Sciences).

 Spots were processed and digested with trypsin (Sigma Aldrich) with minor modification of the procedures of [18]. Tryptic peptides were loaded onto an enrichment column and analyzed by a 75-µm x 150 mm SB-C18 separation column (Agilent Technologies, Santa Clara, CA, USA). Peptides were separated by flow rate at 300 nl per minute, with solvent A (0.2%, v/v formic acid in water) and solvent B (100 % acetonitrile) and the following gradients: at 0, 50, 54, 56 minute after injection with 3%, 35%, 80%, 100% solvent B, respectively. The LC-MS/MS analysis was carried out using an Agilent 1100 HPLC and 6340 ion trap system with MS scan range from 300 to 1,300 m/z and MS/MS by collision-induced dissociation. A 30-second dynamic exclusion was applied to the precursor previously selected for MS/MS twice. Raw data files from LC-MS/MS were searched against NCBInr using Spectrum Mill MS Proteomics Workbench (v03.03.084, Agilent Technologies). The data Extractor utility program detected peaks, assigned precursor charges where possible (for those the charge state was not successfully determined by the software, 2+ to 5+ were considered), filtered MS/MS spectra by quality attributes (spectra with peak number > 4 and sequence tag length > 2 were kept for MS/MS search), centroided the MS/MS spectra, merged nearby MS/MS spectra from the same precursor by default MS/MS similarity criteria and generated peak lists. The peak lists were searched against the database by the following criteria: two missed trypsin cleavages, fixed modification (carbamidomethylation on cysteine), variable modifications (oxidized methionine and pyro-glutamic acid modification at N-terminal glutamines), precursor mass tolerance +/- 2.5 Da, product mass tolerance +/- 0.7 Da. A minimum two peptides were required to conclude identification.

### Phosphatase treatment of oocyte extracts

 Oocyte extracts were prepared as described above except oocytes were homogenized in 10mM HEPES, 100mM KCl, 2mM MgCl_2,_ 50mM sucrose pH to 7.7 with KOH including protease inhibitors. The extracts were centrifuge at 10000g for 20 mins to remove insoluble material. 10µg of extract was treated with 1 unit of calf intestinal alkaline phosphatase (NEB) for 1 hour at room temp. Samples were then analyzed by 2D gel electrophoresis, and the proteins western blotted onto nitrocellulose (Whatman, PROTRAN).

### Injection of Oocytes

 Injection needles were made using a vertical pipette puller (Kopf, model 720). Needles were backfilled with light mineral oil and mounted on a Drummond Nanojet 2 microinjector. 46 nl of samples were routinely injected into oocytes. When oocytes were induced with progesterone they were incubated for 10 min after injection prior to progesterone addition. All metabolite injection studies were repeated 3 or more times and representative results are presented for each experiment. In each experiment, batches of at least 20 oocytes were used for each condition. All experiments yielded similar outcomes, but temporal and dose differences were observed due to inherent batch-to-batch variation.

### ROS Assay

 Typically 8-10 oocytes were incubated in 0.55 ml of the appropriate media in a 24 well plate containing H_2_DCFDA at a final concentration of 30 μM for 1 hour in the dark. Oocytes were collected and washed once with PBS and then lysed in 100 µl of mitochondrial lysis buffer (210 mM mannitol, 60 mM sucrose, 10 mM Hepes (pH 7.5), 10 mM KCl, 5 mM EGTA, 10 mM succinic acid) supplemented with protease and phosphatase inhibitors (Sigma). Platelets and melanosomes were removed by low speed centrifugation (800 X g) at 4°C for 10 min. The mitochondrial fraction was obtained by transferring the supernatant to a second tube and centrifuging (16,000xg) for at 4°C for 10 min. The supernatant was collected and retained as the cytoplasmic fraction. The mitochondrial pellet was washed once with lysis buffer and resuspended in lysis buffer containing 1% Triton X-100. Following resuspension by vortexing the samples were centrifuged for 2 min at room temperature. 20 µl aliquots were transferred in duplicate to a 96 well plate containing 80 µl of lysis buffer per well and incubated at 32°C for up to 80 min. H_2_O_2_ (2.75-176 mM) diluted in mitochondrial lysis buffer was included as a control. After multiple incubation times ranging from 20 to 80 min the plate was read using a Perkin Elmer Victor^3^ 1420 multilabel counter (Fluorescein channel (485/535). 

### Western Blotting

 Samples (20-30 µg) were separated by SDS-PAGE and transferred to nitrocellulose (Whatman, PROTRAN). Blots were blocked with 5% nonfat milk powder in TBS-T (25 mM Tris (pH 7.4), 137 mM NaCl, 2.7 mM KCl, 0.05% Tween-20) and then probed with specific antibodies. Primary antibodies were visualized by incubation with horseradish peroxidase labeled secondary antibodies and ECL reagents (HyGlow, Denville Scientific Inc.). 

## Results

### Comparative proteomic analysis of stage VI oocytes and mature oocytes

 As *Xenopus* oocyte maturation is regulated post transcriptionally through changes to the proteome, we sought to identify differences in the proteome that occur during the maturation of a stage VI oocyte using two-dimensional differential gel electrophoresis (2D-DIGE). Lysates were prepared from surgically isolated stage VI oocytes and stage VI oocytes from the same animal that had been matured by incubation in progesterone until white spot formation (WSF). After dye labeling the different samples were analyzed by 2D-DIGE and 1096 protein spots were assigned. The changes in abundance of these spot were quantified and those with relative abundance changes of 1.3 times or greater between the two samples (greater or lesser) at a confidence of 95% (*p*<0.05) were deemed significant. Within this group 59 were elevated in the matured oocyte sample and 49 were elevated in the oocyte sample, suggesting that this method had detected changes to the proteome during oocyte maturation. The protein in 36 of the spots was identified with 10 increasing in abundance and 26 decreasing during progesterone-stimulated maturation (Table 1).

**Table 1:** List of all protein changes from 2D-DIGE comparison of *X. laevis* stage VI oocyte with progesterone matured stage VI oocytes.

**PROTEIN**	**FOLD CHANGE (oocyte to egg)**	**GROUP**
eEF2	+1.4	Translation
eEF2	+1.4	Translation
eEF1γ	+1.8	Translation
eEF1γ	+4.3	Translation
eEF1γ	-11.8	Translation
eEF1γ	-2.5	Translation
eEF1γ	-2.9	Translation
GRP78	-1.6	Protein folding
GRP78	-1.6	Protein folding
GRP58	-1.4	Protein folding
GRP58	-1.3	Protein folding
Leucine amino peptidase 3	+1.5	Protease
Leucine amino peptidase 3	-1.3	Protease
Leucine amino peptidase 3	-1.4	Protease
Cathepsin D	-1.3	Protease
Cndp dipeptidase 2	-1.6	Protease
Psma2 (proteasome)	-2.9	Protease
Thioredoxin domain containing protein 5	-1.5	REDOX homeostasis
Thioredoxin domain containing protein 5	-1.3	REDOX homeostasis
Peroxiredoxin 4	-1.6	REDOX homeostasis
Peroxiredoxin 6	-1.4	REDOX homeostasis
Perilipin	-1.3	Metabolism
Enolase B	-1.3	Metabolism
Phosphoglycerate mutase (PGA)	+1.4	Metabolism
Aconitase	+1.5	Metabolism
Ribulose-5-phosphate isomerase	-1.4	Metabolism
Phosphoglycerate mutase	-1.4	Metabolism
Enolase A	-1.6	Metabolism
Aldolase C	-1.9	Metabolism
Triose phosphate isomerase	-3.0	Metabolism
Phosphoglucomutase 2	+2.0	Metabolism
Phosphoglycerate kinase 2	-4.9	Metabolism
Triose phosphate isomerase	-2.6	Metabolism
Deoxythymidylate kinase	+1.4	Metabolism
Phosphoglucomutase 2	+1.9	Metabolism
Phosphoglycerate kinase 2	-4.9	Metabolism

**Figure 1:**
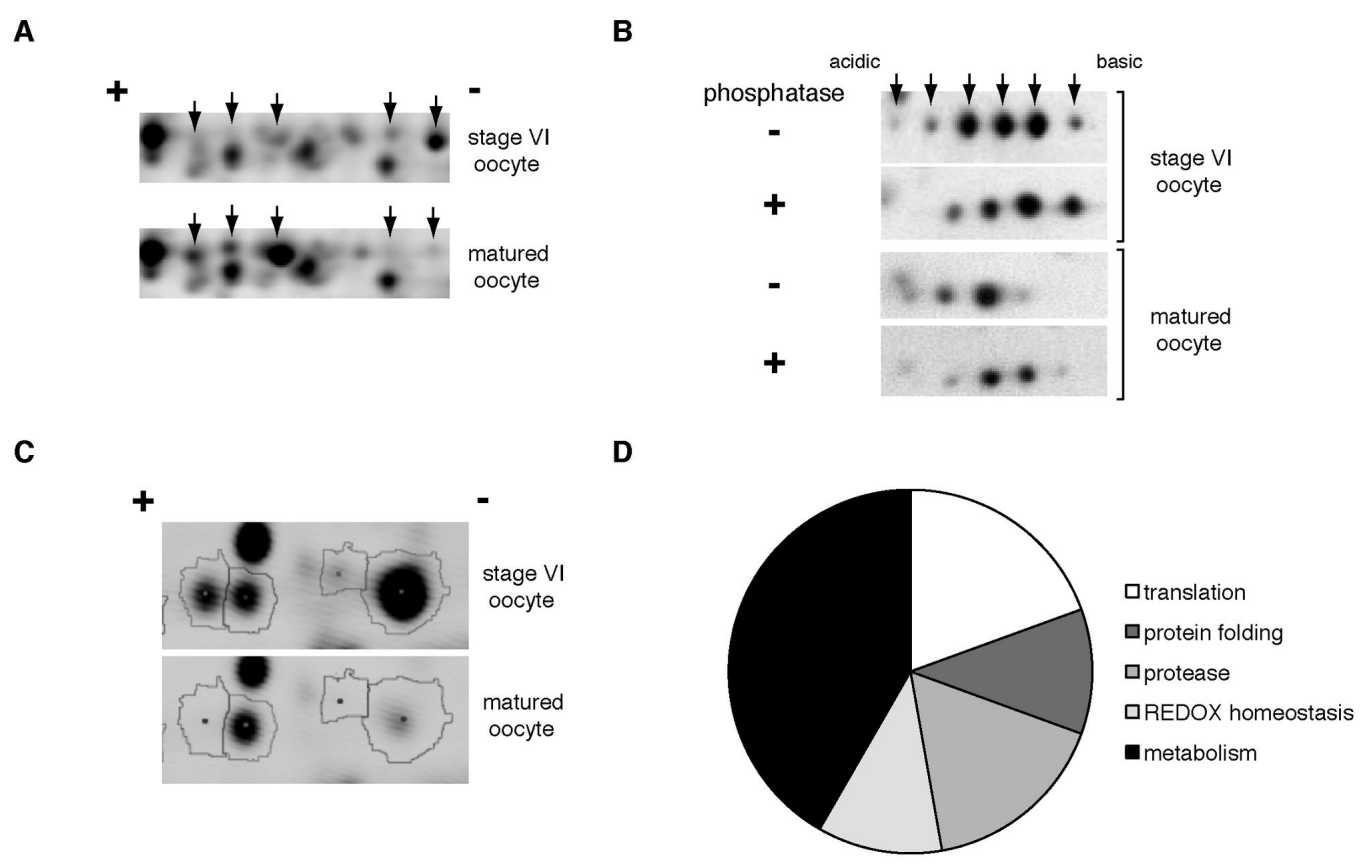
Changes in the maturing oocyte proteome detected by 2D-DIGE. A. Region of a 2D-DIGE gel with arrows pointing to protein spots identified as EF1γ. B. Western blot probing 2D-gels separating stage VI oocyte and egg protein in the presence and absence of alkaline phosphatase treatment. Arrows point to the different EF1γ isoforms. C. A region of a 2D-DIGE gel with the protein spot areas of spots identified as enolase demonstrating changes in isoform abundance between stage VI oocytes and eggs. D. Pie chart of the relative proportion of different cellular pathways affected by changes in proteins levels during oocyte maturation as detected in 2D-DIGE experiments.

A positive value indicates an increase in protein levels in the matured oocyte compared to the stage VI oocyte and negative values indicate decrease levels in the matured oocyte. Some proteins formed multiple spots. This is probably dues to different post-translational modifications (see EF1γ in Figure 1A, B) or the potential presence of multiple isoforms in the egg extract in the case of enolase.

 Of those positive identifications, 10 proteins had multiple spots. In the case of enolase the multiple spots (Figure 1C) reflect different isoforms. However, in the case of eEF1γ the multiple spots reflect changes in post-translational modification (Figure 1A, B). eEF1γ is known to be a phospho-protein. To determine if the multiple eEF1γ positive spots were due to differential phosphorylation, we treated stage VI and matured oocyte extracts with calf intestinal alkaline phosphatase and analyzed the extracts on 2D gels and western blotting to detect eEF1γ(Figure 1B). Phosphatase treated extracts had an eEF1γ spot distribution biased toward the basic end of the isoelectric focusing gradient. In contrast, untreated extracts had an eEF1γ spot distribution biased toward acidic end of the isoelectric focusing gradient, reflective of a more phosphorylated state. In addition, eEF1γ spots were distributed with a greater bias to the acidic end in matured extracts when compared to stage VI oocyte extracts, suggesting that during maturation eEF1γ became increasingly phosphorylated (Figure 1B).

**Figure 2:**
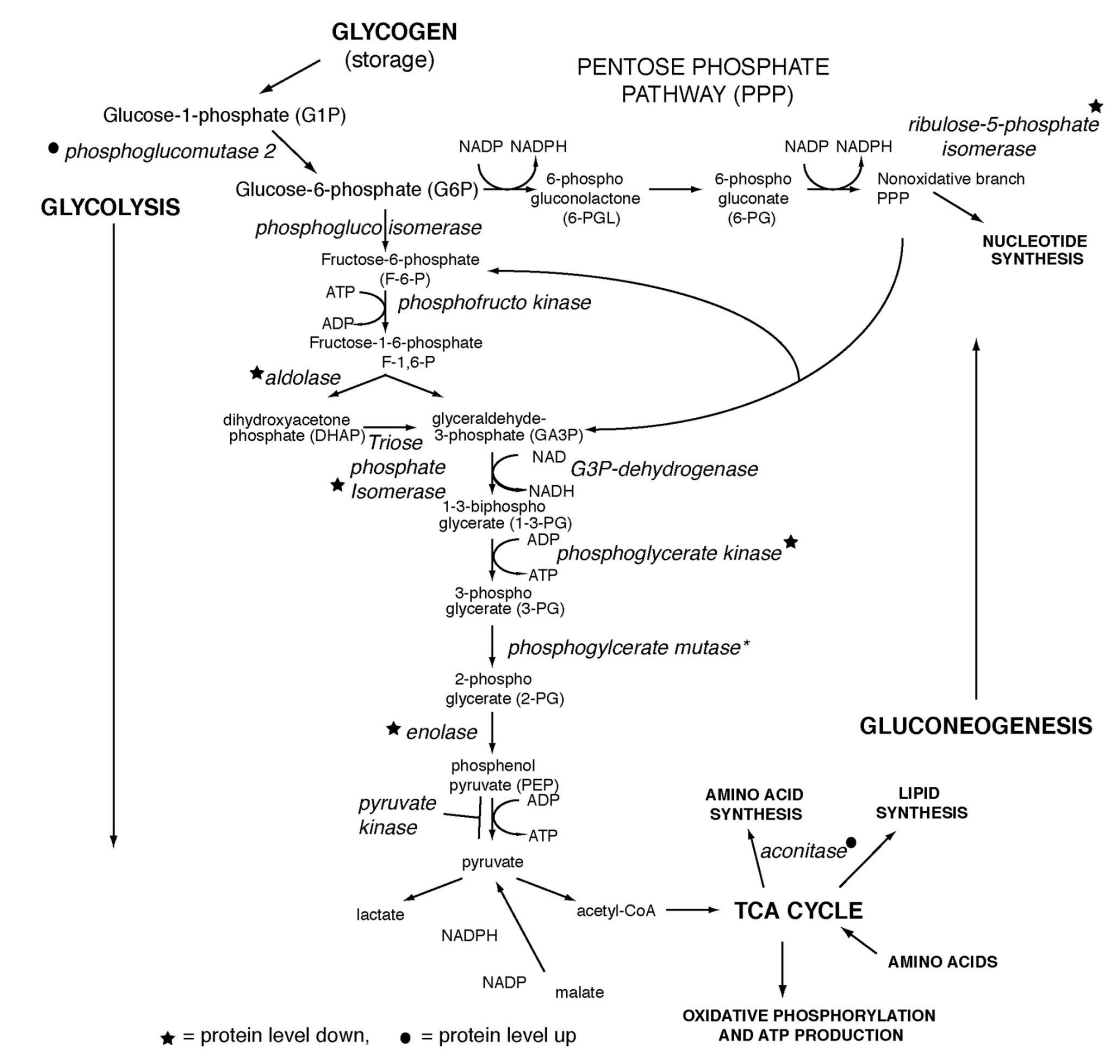
Glycolytic and related metabolic pathways. Black circles denote enzymes that become more abundant during maturation and black stars denote enzymes that decreased in abundance during maturation. The asterisk marks an enzyme, phosphoglycerate mutase that had one spot increase and another decrease by the same level during maturation suggesting the protein level remained constant, but the protein became modified during oocyte maturation.

 The proteome changes were restricted to a small number of biological pathways: translation, protein folding, REDOX regulation, proteolysis and metabolism (Figure 1D). Of those groups the metabolism group was the largest and concentrated in the glycolytic and immediately related pathways of the tricarboxylic acid cycle (TCA cycle) and PPP (Figure 2).

### Glucose-6-phosphate induces apoptosis in maturing oocytes

**Figure 3:**
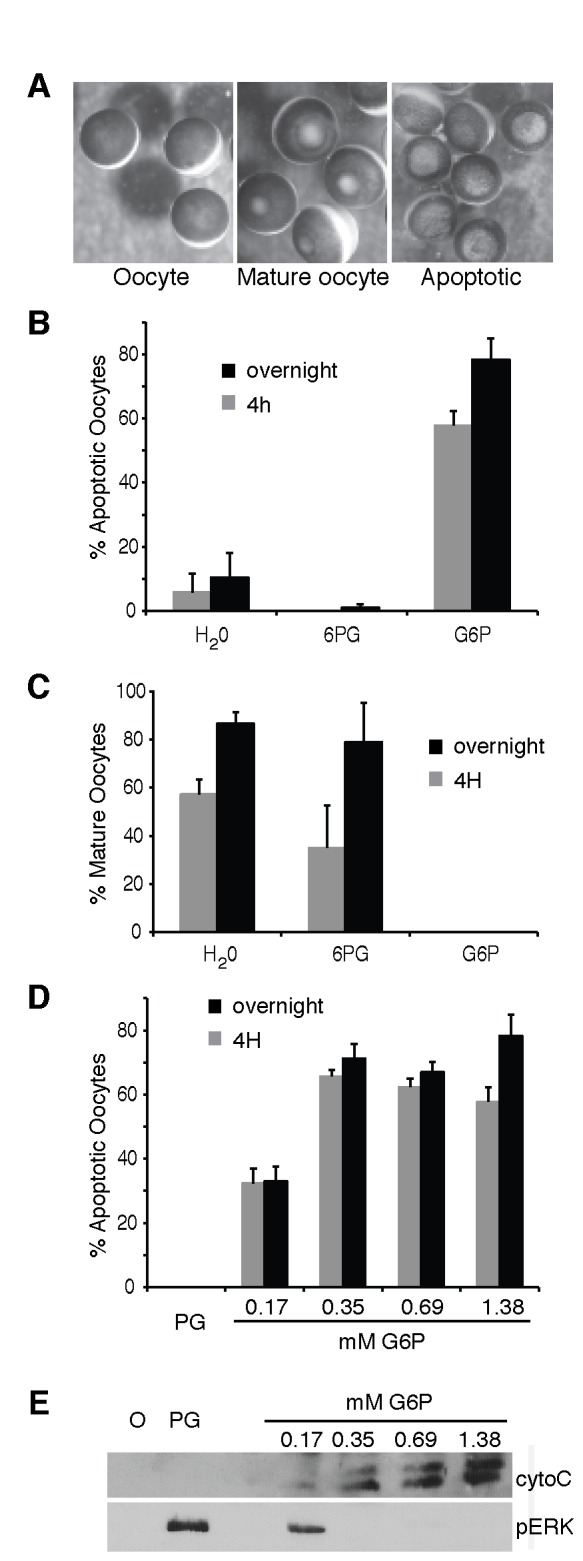
G6P injection induces apoptosis in *X. laevis* oocytes is situ. A. The three phenotypes scored were oocyte, mature oocyte and apoptotic oocyte. B. Oocytes were injected with H_2_O, G6P or 6PG (intracellular concentration elevated by 1.38mM) and monitored for apoptosis 4 hours or overnight post progesterone addition. The results presented are representative of at least 3 independent experiments. C. Same as (B) except the oocytes were scored for maturation. D. Oocytes were injected of G6P solutions to elevate metabolite concentrations as indicated. Oocytes were monitored and scored for apoptosis at the indicated times. E. Oocytes were injected with metabolite, incubated in progesterone then collected and a post-mitochondrial supernatant was prepared and analyzed by Western blotting with antibodies specific for cytochrome C (cyto C) or phospho-ERK (pERK). O = uninjected stage VI oocyte. Error bars are +SEM.

 To assess if glycolytic intermediates have a role in oocyte maturation, we injected the different glycolytic metabolites into surgically isolated stage VI oocytes. The bias of glucose metabolism in the oocyte is in the glycogenic direction [13,19] although there is evidence that glycolysis is also active [20]. First we injected glucose-6-phosphate (G6P), elevating the oocyte G6P concentration by 1.38 mM, as this is the final product of gluconeogenesis. The intracellular concentration of the G6P in the oocyte is reported range from 0.25 mM [13] to 1 mM [21]. At around 4 hours after incubation in progesterone, oocytes injected with G6P formed a much larger white spot at the animal pole than was typical of WSF during oocyte maturation (Figure 3A). This larger white spot is characteristic of an oocyte becoming apoptotic [4,6]. On average about 60% of the oocytes from different batches taken from different animals became apoptotic at 4 hours after G6P injection, rising to 80% of oocytes being apoptotic when left overnight (Figure 3B). In contrast, only 6% of oocytes became apoptotic 4 hours after the injection of the same volume of water, rising to 10% when left overnight. Injection of 6-phosphogluconate (6PG), which lies downstream of G6P in the PPP, did not stimulate apoptosis (Figure 3B). While G6P injection induced apoptosis it inhibited maturation with no oocyte maturation being observed after extended overnight incubation in progesterone (Figure 3C). In contrast, injection of water or 6PG did not prevent oocyte maturation (Figure 3C). As a range of G6P concentrations have been determined in the oocyte [13,21], we tested different concentrations of G6P from 0.17mM to 1.38mM. Increasing concentrations of G6P caused a greater percentage of oocytes to develop an apoptotic phenotype (Figure 3D).

 An alternative method to follow apoptosis is to follow the release of cytochrome C into the cytosol. Using this method we confirmed the visual apoptotic phenotype described above, finding that increasing concentrations of G6P caused release of cytochrome C into the cytosol. Likewise increasing concentrations of G6P prevented oocyte maturation as seen by a failure to activate the MAP kinase family member ERK, an indicator of nuclear oocyte maturation (Figure 3E).

### PPP intermediates and products suppress the apoptotic activity of G6P

**Figure 4:**
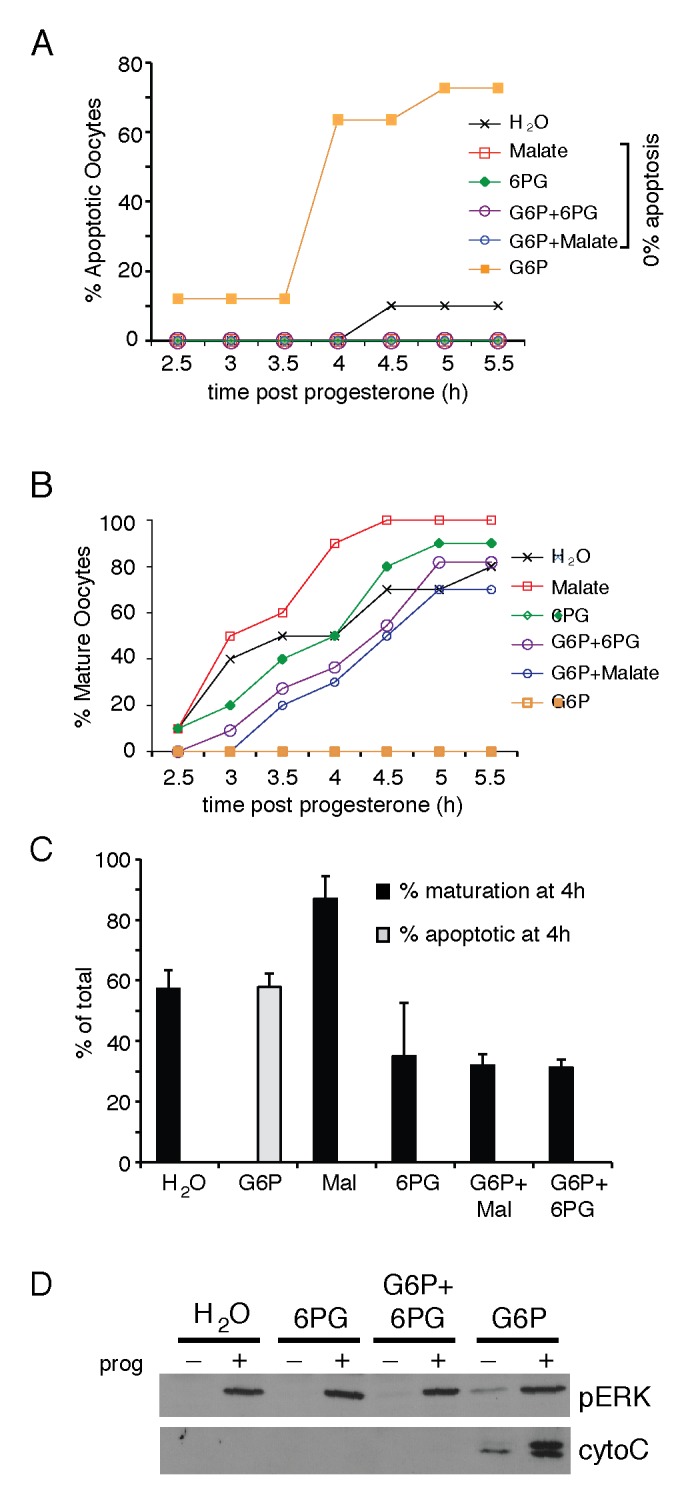
NADPH generating metabolites inhibit apoptosis induced by G6P. Representative experiment of oocytes injected with G6P alone or in combination with malate or 6PG (1.38 mM elevation in the intracellular concentration of each metabolite), then treated with progesterone. The oocytes were monitored for apoptosis in A, or maturation in B at the indicated time points post progesterone treatment. C. As (A) and (B) but combined analysis of at least 3 batches of oocytes from different animals. Error bars are +SEM. D. Cytoplasmic extracts prepared from the oocytes incubated in the presence or absence or progesterone (prog) were analyzed by Western blotting with antibodies specific for cytochrome C (cyto C) or phospho-ERK (pERK).

 Our data differs from previous *in vitro* studies in *Xenopus* egg extracts where G6P metabolism through the PPP suppressed apoptosis [4]. Our data also demonstrate that 6PG does not interfere with oocyte maturation and suggests that G6P in *Xenopus* oocytes may not be primarily metabolized through the PPP, an observation consistent with those made in maturing mouse oocytes [22]. In *Xenopus* egg extracts, NADPH production through the PPP prevents the activation of Caspase 2, thereby preventing the activation of apoptosis [4]. To determine whether NADPH production had a protective effect on maturing oocytes, we injected 6PG or malate into maturing oocytes in the presence and absence of elevated G6P. 6PG can be converted to ribulose-5-phosphate and generate NADPH in the PPP and malate can be converted to pyruvate and generate NADPH [12,13]. Co-injection of malate or 6PG with G6P effectively inhibited apoptosis induced by G6P and also restored progesterone-induced maturation (Figure 4A, C). Indeed, malate injection enhanced the maturation rate of oocytes in individual experiments (Figure 4B). As oocytes from different animals at different times of the year mature at different rates, representative data from individual animals is shown (Figure 4A, B), rather than averages of many animals [4]. However, this data is further supported when findings from multiple animals are compared at a single fixed time point, 4 hours (Figure 4C). The anti-apoptotic activity of malate and 6PG was further assessed by analysis of cytochrome C release into the cytoplasm. Co-injection of malate or 6PG with G6P prevented the release of cytochrome C into the cytoplasm observed upon injection of G6P alone. (Figure 4D). These data suggest that G6P is not being metabolized primarily through the PPP in maturing oocytes and its apoptotic inducing activity can be neutralized by NADPH production.

### GA3P and PEP induce apoptosis in maturing oocytes

**Figure 5:**
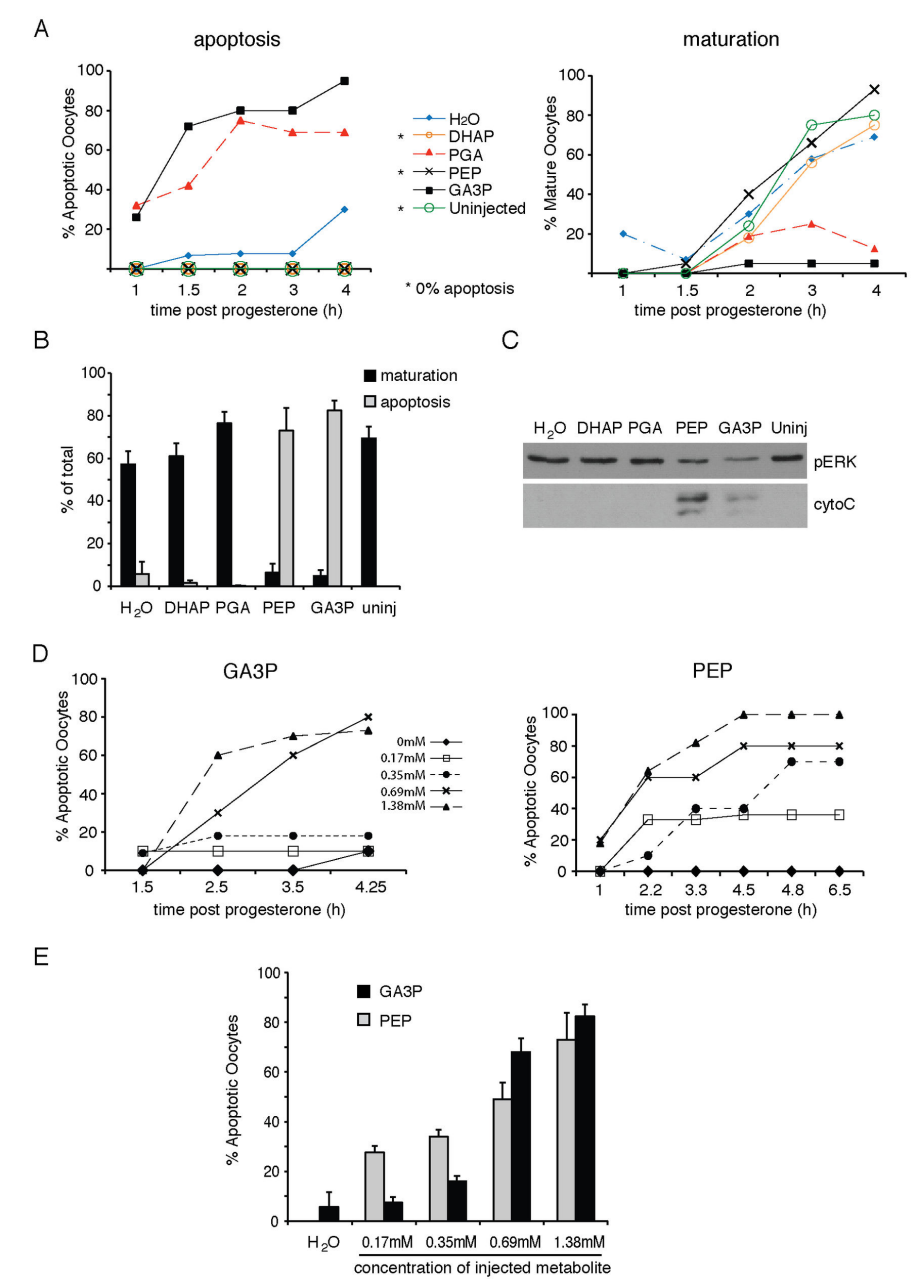
Glycolytic intermediates regulate apoptosis and maturation *in* *situ*. A. Representative results from one batch of oocytes injected with the indicated glycolytic intermediates, induced with progesterone, and scored at the indicated times for apoptosis or maturation. B. As (A), but combined analysis at the 4 hour time point after progesterone treatment of at least 3 batches of oocytes from different animals. Error bars are +SEM. C. Oocytes were lysed and cytoplasmic extracts were analyzed by Western blotting with antibodies specific for phospho-ERK (pERK), or cytochrome C (cyto C). D. Representative results from one batch of *X. laevis* oocytes injected with GA3P or PEP at the indicated intracellular concentrations of injected metabolite. The oocytes were monitored and scored for apoptosis at the indicated time periods post progesterone addition. E. As (D), but combined analysis of at least 3 batches of oocytes from different animals at 4 hours after progesterone treatment. Error bars are +SEM.

 As G6P is inefficiently metabolized through PPP, it is possible that downstream metabolites of G6P maybe responsible for its apoptotic activity. Therefore, we tested different glycolytic intermediates in this assay including dihydroxyacetone phosphate (DHAP), glyceraldehyde-3-phosphate (GA3P), 2-phosphoglycerate (2-PGA) and phosphoenolpyruvate (PEP). Following injection (intracellular concentration of each injected metabolite was 1.38mM) the oocytes were induced with progesterone and monitored for both maturation and apoptosis. These metabolites had dramatically different effects. Injection of GA3P or PEP into oocytes induced an apoptotic phenotype, while 2-PGA and DHAP, an isomer of GA3P had no observable effect on either maturation or apoptosis (Figure 5A, B). The degree of apoptosis observed phenotypically was confirmed by monitoring the level of cytoplasmic cytochrome C. GA3P and PEP induced release of cytochrome C into the cytoplasm while the other metabolites did not (Figure 5C). The degree of apoptosis induced by PEP and GA3P was dose dependent (Figure 5D, E). These data suggest that elevated levels of three glycolytic intermediaries, G6P, GA3P and PEP, induces apoptosis in maturing oocytes.

### NADPH production suppresses the apoptotic activity of Glycolytic Intermediates

**Figure 6:**
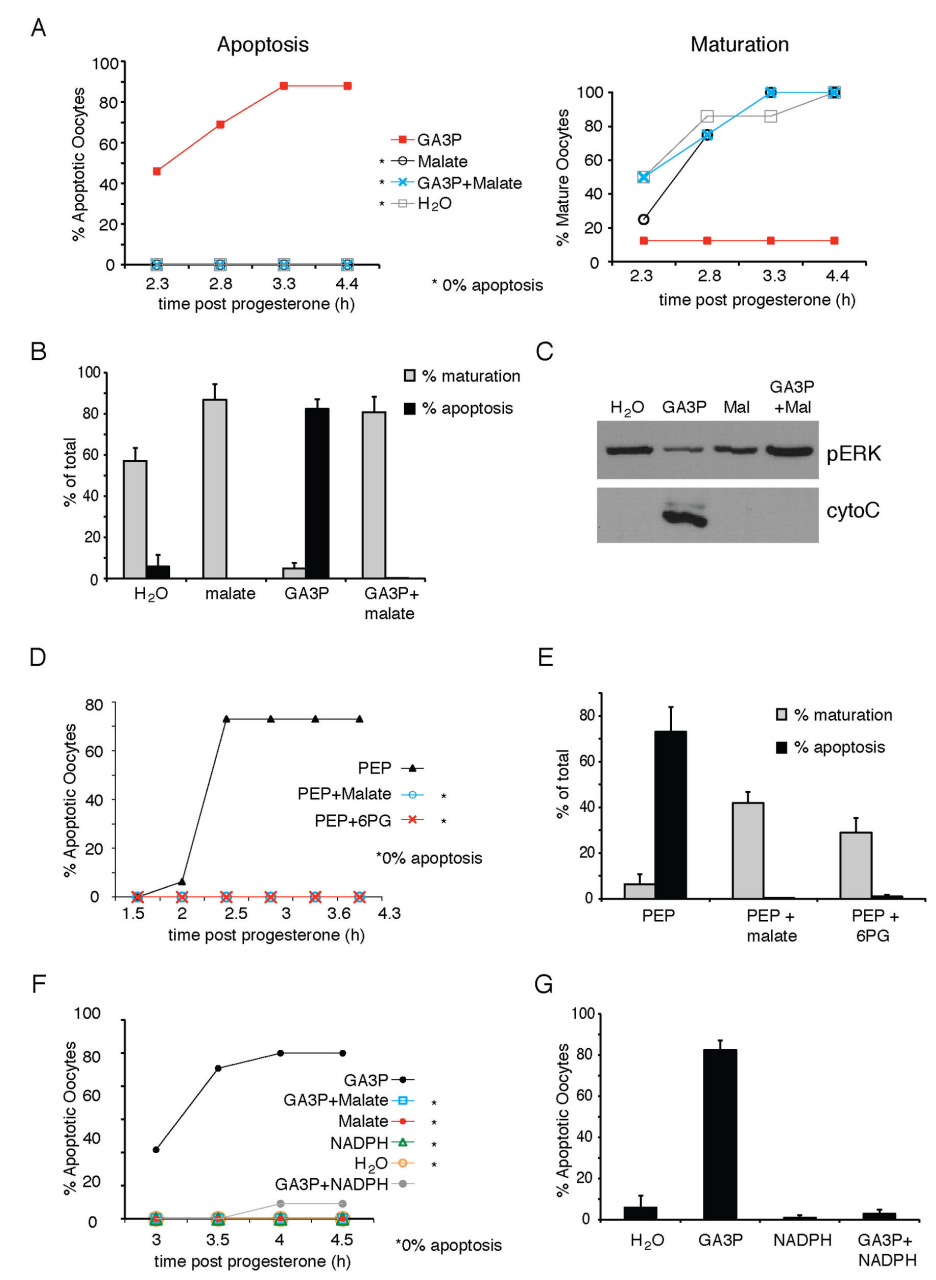
NADPH generating metabolites inhibit apoptosis induced by glycolytic intermediates. A. Representative results from one batch of oocytes injected with GA3P either alone or in combination with malate (metabolite concentration elevated by 1.38 mM). At the indicated time points post progesterone stimulation the oocytes and scored for apoptosis or maturation. B. As (A), but combined analysis at 4 hours after progesterone treatment of at least 3 batches of oocytes from different animals. Error bars are +SEM. C. Oocytes were collected and cytoplasmic extracts were prepared and analyzed by Western blotting with antibodies specific for cytochrome C (cytoC) or phospho-ERK (pERK). D. Representative results from one batch of oocytes injected with PEP alone or in combination with malate or 6PG (each metabolite concentration elevated by 1.38 mM). Oocytes were monitored and scored for apoptosis at the indicated time point post-progesterone addition. E. As (D) but combined analysis at 4 hours after progesterone treatment of at least 3 batches of oocytes from different animals scored for maturation and apoptosis. Error bars are +SEM. F. Representative results from one batch of oocytes injected with GA3P alone or in combination with malate or NADPH (each metabolite concentration elevated by 1.38 mM). Following progesterone addition the oocytes were scored for apoptosis at the indicated time points. G. As (F) but combined analysis at 4 hours after progesterone treatment of at least 3 batches of oocytes from different animals scored for maturation and apoptosis. Error bars are +SEM.

 We next tested the capacity of reagents found to prevent G6P induced apoptosis to suppress the activity of the apoptosis inducing glycolytic intermediates. Co-injecting malate with GA3P (intracellular concentration was elevated by 1.38 mM for each) effectively inhibited the apoptotic activity of GA3P (Figure 6A). Furthermore co-injection of malate restored progesterone-induced maturation (Figure 6A, C). The level of cytoplasmic cytochrome C and degree of ERK phosphorylation reflected the phenotypes observed (Figure 6C). Similar results were obtained when PEP was used as the apoptosis inducer with 6PG or malate co-injected (intracellular concentration was elevated by 1.38 mM for each. Figure 6C-E). In addition, direct injection of NADPH effectively inhibited apoptosis induced by GA3P (Figure 6F, G) and PEP (data not shown).

### G6P, GA3P and PEP increase the level of Reactive Oxygen Species in oocytes

**Figure 7:**
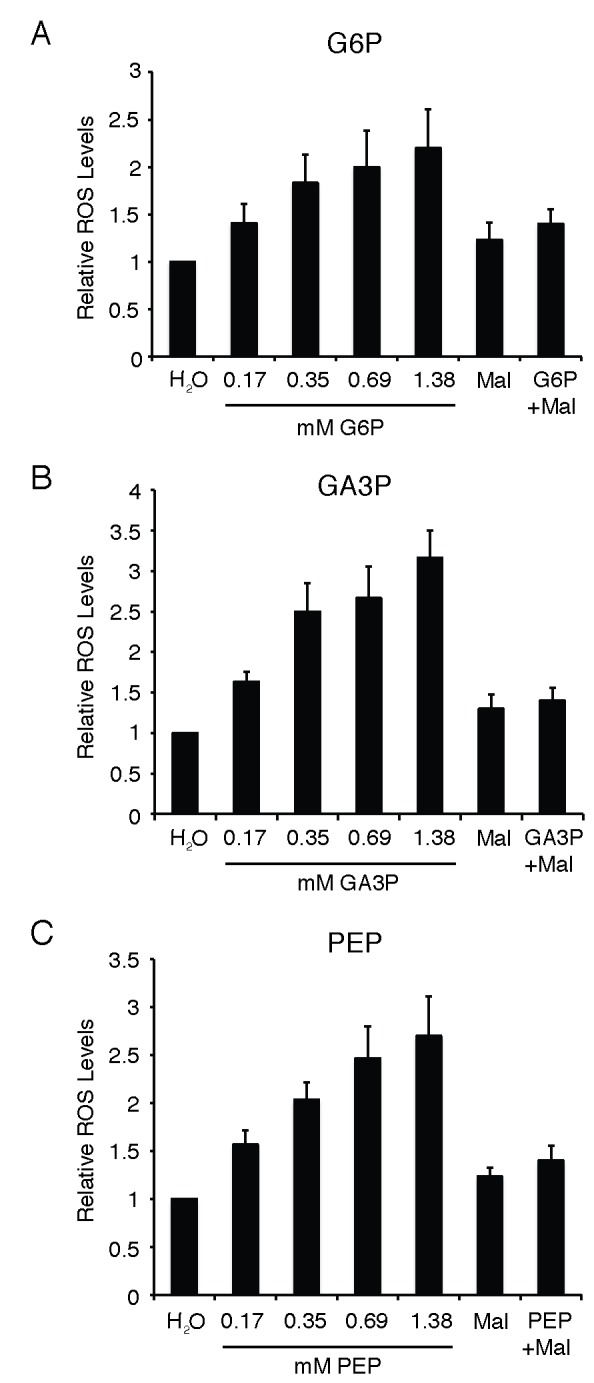
Apoptosis inducing metabolites increase intracellular ROS levels. A. Oocytes were injected with different doses of G6P (elevating the intracellular metabolite concentration by 0.17-1.38 mM), malate (1.38 mM), or a combination of malate and G6P (1.38 mM each), incubated with progesterone then monitored until apoptosis was observed in the GA3P injected samples (4 hours post injection). Oocytes were lysed and a mitochondrial pellet prepared for analysis of mitochondrial ROS levels. Data compiled form at least 3 different batched of oocytes form 3 different animals. Error bars are +SEM. B. Same as (A) except that GA3P was used as the apoptosis-inducing reagent. As in (A) malate was injected to a final concentration of 1.38mM and when injected in combination with GA3P, both metabolites were injected to a concentration of 1.38mM. Same as (A) except PEP was used as the apoptosis inducing metabolite. As is (A) and (B) malate was injected to a final concentration of 1.38mM and when injected in combination with PEP, both metabolites were injected to a concentration of 1.38mM.

 NADPH can influence the level of reactive oxygen species (ROS) in cells thereby affecting different aspects of cellular physiology including apoptosis and mitotic progression [16]. To determine if the apoptotic and maturation effects we observed with injected metabolites involved changes in ROS levels, we examined mitochondrial ROS levels during the different conditions employed. G6P induced a dose-dependent increase in ROS levels, which was effectively countered by including malate (Figure 7A). GA3P also induced a dose dependent increase in apoptosis and mitochondrial ROS levels (Figure 7B). Again when malate was co-injected as an anti-apoptosis reagent with GA3P (intracellular concentration was 1.38 mM for each), the GA3P induced rise in ROS was effectively mitigated. Malate alone did not affect mitochondrial ROS levels. PEP also generated a dose dependent increase in ROS levels in oocytes coincident with induction of apoptosis (Figure 7C). These data suggest that the glycolytic intermediates G6P, GA3P and PEP reduce oocyte viability by elevating oocyte ROS.

## Discussion

By using comparative proteomics we identified changes in the proteome that occur during *Xenopus laevis* oocyte maturation. As oocyte maturation is regulated by translation, changes to the translation machinery were expected. However, the extent of changes to enzymes in the glycolytic pathway were unexpected. Based on these findings we show that changes in oocyte metabolism during oocyte maturation can affect oocyte survival by regulating the oocyte susceptibility to ROS mediated apoptosis.

In *Xenopus* the fully developed stage VI oocytes is poised to reenter the meiotic cell cycle. Following hormonal stimulation it may proceed through meiosis and subsequently be amenable to fertilization. Alternatively, the oocyte can be eliminated via apoptosis if conditions, either internal or external, are not appropriate. The availability of adequate stored nutrients is one factor that may have a role in the outcome. Nutrient resources and availability may provide a regulatory switch that determines the destiny of the oocyte [4,7]. However, our data further suggests that metabolic intermediaries can also regulate apoptosis and may allow a more exquisite regulation of oocyte survival than previously thought.

Our *in situ* study corroborates a previous *in vitro* study suggesting that the generation of NADPH can stave off oocyte apoptosis. Using the *in vitro* egg extract system, Nutt and co-workers characterized the molecular mechanism by which reduced NADPH levels may induce apoptosis [4,23]. We assume that the same downstream molecular mechanisms are at play in the oocytes in our study that undergo apoptosis and fail to mature. However, the two studies do differ in the mechanism by which apoptosis occurs. The *in vitro* studies of Nutt and co-workers suggest that a general depletion of nutrient stockpiles in the egg and oocyte, in particular G6P by the PPP, accounts for the depletion of NADPH. In contrast, our current study suggests G6P is inefficiently metabolized through the PPP in maturing oocyte, a finding consistent with observations in maturing mouse oocytes [22]. Our data further suggests that specific glycolytic metabolites can induce apoptosis and therefore continued metabolism may generate deleterious compounds that over time could cause apoptosis and a reduction in oocyte viability. Why these studies should differ in some of their findings especially in the opposite effects of G6P and GA3P is not clear. It is possible that the mature egg, used to make extract for the *in vitro* studies [4,23], is significantly different regarding metabolite composition relative to the oocyte regardless of the *in vitro* versus *in situ* circumstances and therefore inferences made from experiments using eggs in relation to oocytes may not be valid. Previous studies found no significant differences in the metabolic pattern in oocyte and eggs [13]. However, these studies compared stage VI oocytes and fertilized eggs and did not analyze progesterone stimulated maturing oocytes. During oocyte maturation oxygen consumption and reduced forms of the pyridine nucleotides increase [3,21]. Such metabolic alterations could contribute to the differences observed. Another critical factor could be the preparation of the extract, which involves crushing the eggs and taking only the cytoplasmic fraction for further analysis whilst disregarding other fractions that include a variety of intracellular membranes [24]. It is distinctly possible that these membrane fractions contain critical activities for metabolism and therefore posses key activities for oocyte maturation and survival.

As carbon flux through the PPP prevents apoptosis, one mechanism by which apoptosis could proceed is through a reduction in activity of the PPP. Evidence exists for such a mechanism in mouse oocytes. The activity of the enzyme G6PDH, the first enzyme that commits carbon to the PPP, was found to decrease in oocytes isolated from aged mice as compared to younger mice [25]. The consequence of this could be to reduce the amount of carbon flux through the PPP thereby reducing the levels of NADPH within the oocyte and causing a rise in ROS levels leading to apoptosis. How this reduction in activity occurs is unknown. One mechanism could be to reduce the levels of the enzymes. In our study ribulose-5-phosphate isomerase, an enzyme in the PPP, became less abundant during oocyte maturation. In mice, microarray studies revealed that the transcripts of many TCA enzymes were degraded during oocyte maturation [25]. These observations suggest that oocytes could regulate metabolic pathways by altering the levels of enzymes during maturation. A potential consequence of this could be to change carbon flux during maturation, thereby regulating oocyte and egg lifespan.

Careful regulation of NADPH levels is critical as increased levels of NADPH could also produce an imbalance in the redox environment. NADPH can, on one hand, fuel an increase in ROS levels through NADPH oxidase or, on the other hand, provide reducing power to enzymes such as glutathione peroxidase and catalase that limit ROS production [17]. We observed a marked increase in ROS levels in the oocyte following injection of apoptosis inducing metabolites that was effectively countered by all metabolites that inhibit apoptosis. Under these circumstances NADPH or inducers of NADPH clearly inhibit the ROS increase associated with apoptosis induction by these metabolites. A similar anti-apoptotic effect was observed upon pretreatment of oocytes with GSH-ethyl ester which prevented increased ROS levels associated with injection of neutral sphingomyelinase [5]. In addition, the products of ornithine decarboxylase (ODC) are essential for the proper maintenance of ROS levels during the cytoplasmic maturation phase of *Xenopus* oocytes [6]. When ODC levels are reduced the oocyte experiences an increase in ROS leading to apoptotic cell death during the “cytoplasmic maturation” component of the meiotic cell cycle. Together these data reveal an important role for metabolic regulation of ROS in the maintenance of oocyte and egg integrity and survival. 

Our findings may also be applicable to the consequences of ageing mammalian oocytes [26] and tumors where changes in metabolic flux have been described (reviewed in 27,28), and where an active PPP is required to prevent apoptosis in cancer cells [29] and neurons [30]. Understanding the regulation of cellular metabolism is crucial to understanding the physiological needs of different cell types at different points in their life and in different disease states. It is not just the direction of carbon flux through these pathways in different conditions that must to be determined, but also the role of individual metabolites. Furthermore, the roles of many of the enzymes needs to be further studied as increasingly they are found to have multiple functions. The flexibility of the *Xenopus* oocyte / egg model systems and the ability to perform single cell biochemistry should prove a powerful tool in dissecting the role and regulation of these pathways.
